# Mouse oocytes carrying metacentric Robertsonian chromosomes have fewer crossover sites and higher aneuploidy rates than oocytes carrying acrocentric chromosomes alone

**DOI:** 10.1038/s41598-022-16175-6

**Published:** 2022-07-14

**Authors:** Parinaz Kazemi, Teruko Taketo

**Affiliations:** 1 Department of Biology, McGill University, Montreal, QC H3A 1B1 Canada; 2 Department of Surgery, McGill University, RI-MUHC, Montreal, QC H4A 3J1 Canada; 3 Department of Obstetrics/Gynecology, McGill University, RI-MUHC, Montreal, QC H4A 3J1 Canada

**Keywords:** Cell biology, Developmental biology, Genetics

## Abstract

Meiotic homologous recombination during fetal development dictates proper chromosome segregation in adult mammalian oocytes. Successful homologous synapsis and recombination during Meiotic Prophase I (MPI) depends on telomere-led chromosome movement along the nuclear envelope. In mice, all chromosomes are acrocentric, while other mammalian species carry a mixture of acrocentric and metacentric chromosomes. Such differences in telomeric structures may explain the exceptionally low aneuploidy rates in mice. Here, we tested whether the presence of metacentric chromosomes carrying Robertsonian translocations (RbT) affects the rate of homologous recombination or aneuploidy. We found a delay in MPI progression in RbT-carrier vs. wild-type (WT) fetal ovaries. Furthermore, resolution of distal telomere clusters, associated with synapsis initiation, was delayed and centromeric telomere clusters persisted until later MPI substages in RbT-carrier oocytes compared to WT oocytes. When chromosomes fully synapsed, higher percentages of RbT-carrier oocytes harbored at least one chromosome pair lacking MLH1 foci, which indicate crossover sites, compared to WT oocytes. Aneuploidy rates in ovulated eggs were also higher in RbT-carrier females than in WT females. In conclusion, the presence of metacentric chromosomes among acrocentric chromosomes in mouse oocytes delays MPI progression and reduces the efficiency of homologous crossover, resulting in a higher frequency of aneuploidy.

## Introduction

Aneuploidy is the leading genetic cause of pregnancy loss and congenital disorders in humans^1^. It is well documented that most cases of aneuploidy originate from the female germline, and their frequency increases dramatically with maternal age over 35 years^1–5^. The aneuploidy rate is also high in the oocytes of teen-agers, thus forming a U-shaped curve during the female reproductive life^6^. The aneuploidy rates in the eggs of other mammalian species such as rabbit, horse and pig are reported to be over 10% at reproductive ages^7–9^. By contrast, the aneuploidy rate is exceptionally low in the mouse (*Mus musculus*), less than 5% in most strains^10–13^. Although mice have been extensively used as animal models for studying the maternal age effect on the aneuploidy rate in oocytes^11,14–17^, the fundamental difference in the aneuploidy rates between humans and mice has seldom been addressed.

In mammalian oocytes, correct segregation of chromosomes at the first meiotic division depends on the presence and position of covalent linkages (crossovers) between homologous chromosomes^18–21^, which have been established in the fetal life. Most, if not all, primordial germ cells enter meiosis to become oocytes and reach the end of Meiotic Prophase I (MPI) before birth. Meiotic recombination, a prerequisite for crossover, is made possible by the coordinated formation and repair of numerous double-strand breaks (DSBs) along DNAs. A subset of DNA sites (hotspots) are designated for DSBs by a meiosis-specific histone lysine methyltransferase, PRDM9^22–25^. Homologous chromosomes are aligned by sequential assembly of synaptonemal complex, which is composed of axial elements, transverse filaments and central elements^26–29^. As the synapsis between homologues chromosomes extends, DSBs are repaired to allow for the formation of recombination intermediates. While 90% of DSBs are repaired to restore the original strands, 10% undergo strand exchange, resulting in crossovers. Stabilization of the crossover depends on the MutL protein homologues MLH1 and MLH3^30–32^. At the end of MPI, the synaptonemal complex is disassembled while the homologous chromosomes are held together at the crossover sites^33–35^.

Homologous chromosome search and synapsis involves meiosis-specific chromosome movement. At early MPI, telomeres are anchored to the nuclear envelope (NE) and play a vital role in driving chromosome movement along NE, facilitating homolog juxtaposition and resolution of unfavorable entanglements between non-homologous chromosomes^36–39^. In mouse oocytes, all chromosomes are acrocentric, having the centromere adjacent to one telomeric end (sub-centromeric telomere; C-telomere) and away from the other end (distal telomere; D-telomere). We have previously reported that the two telomeric ends occupy distinct territories during homologous synapsis in oocytes; C-telomeres persist in clusters and undergo synapsis near the completion of synaptonemal complex formation, while D-telomere clusters dissolve earlier and preferentially initiate synapsis^40^. Since other mammalian species have a mixture of acrocentric and metacentric chromosomes, we questioned whether the all-acrocentric chromosomes of mouse oocytes enable more efficient meiotic recombination and crossover, resulting in lower aneuploidy rates.

To address this question, we used mice carrying Robertsonian translocation (RbT) chromosomes, which are metacentric. RbT chromosomes are formed by fusion of two acrocentric chromosomes at their centromeres. Although telomeric sequences of the short arms and a part of the centromeres have been lost, no essential genes are affected by this fusion^41,42^. RbT are the most common balanced chromosomal rearrangement in humans, observed in about 1 of 1000 live births^43^. In the mouse, RbT translocation has resulted in more than 40 different chromosomal races in wild populations, ranging from 2n = 40 to 2n = 22^42,44,45^. In this study, we chose two mouse strains, CByJ.RBF-Rb(8.12)5Bnr (Rb5) and RBF/DnJ(1.3; 8.12; 9.14) (RBF), carrying one and three RbT chromosomes in homozygosity with 2n = 38 and 2n = 34, respectively, and asked (1) how the RbT chromosomes behave and (2) whether the presence of metacentric chromosomes affects acrocentric chromosomes in their homologous synapsis and recombination in oocytes during MPI progression. We used the BALB/cByJ (BALB/c) strain, which shares the genetic background with Rb5, as the wild-type (WT) strain for comparisons.

## Results

### Delay in MPI progression in the oocytes carrying RbT

**Figure 1:**
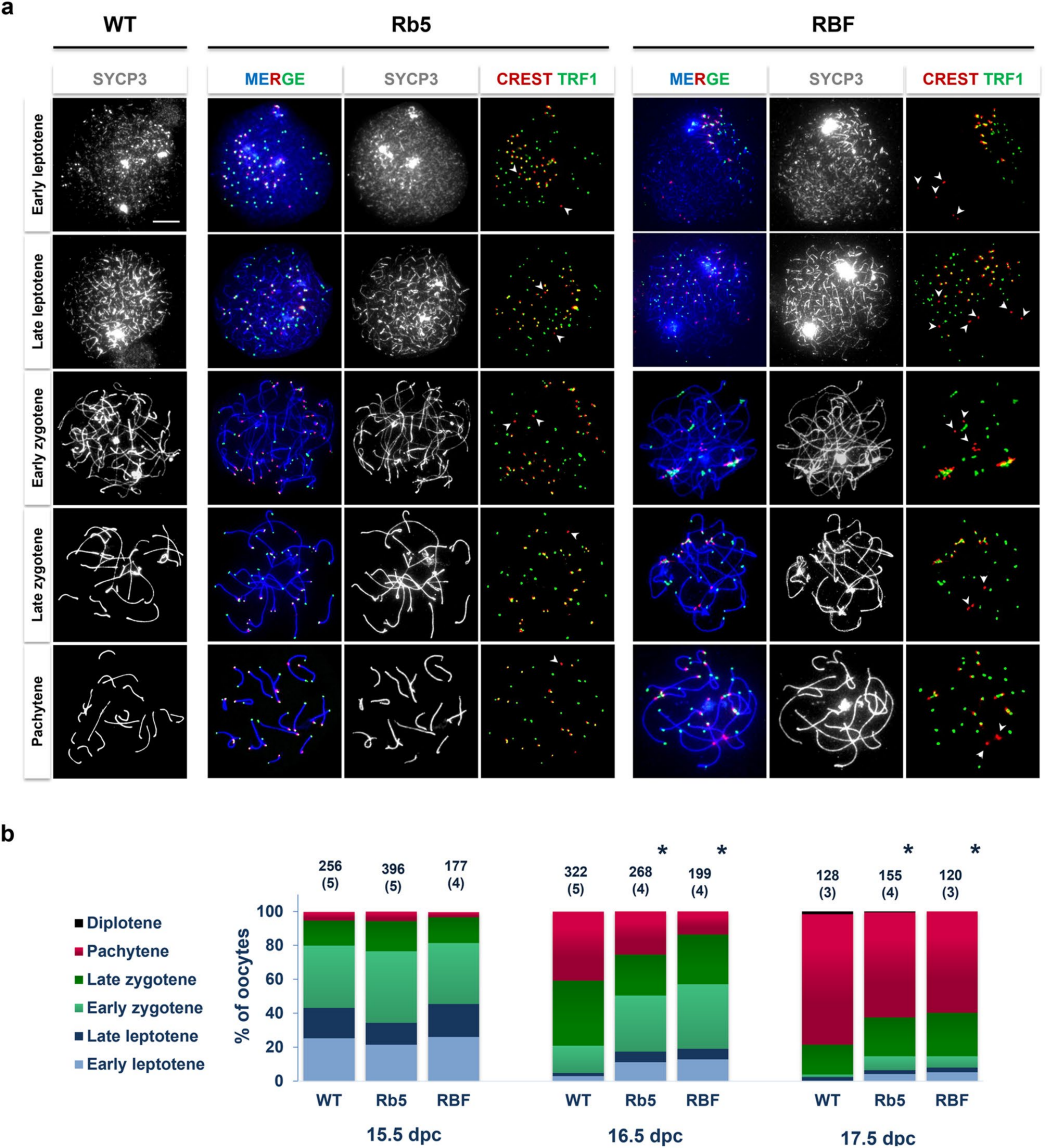
MPI progression in the oocytes of RbT-carrier and WT mouse ovaries at 15.5–17.5 dpc. (**a**) MPI substages identified by SYCP3 immunofluorescence (IF) staining. In Rb5 and RBF oocytes, RbT-centromeres (indicated by arrowheads) were identified by IF-staining of centromeres with CREST antibody (red) without adjacent telomeres with anti-TRF1 antibody (green). C- and D-telomeres were also distinguished by IF-staining of TRF1 with and without adjacent CREST staining, respectively. Scale bar 10 μm. (**b**) Percentages of oocytes at progressive MPI substages in the ovaries of each genotype. The number of examined oocytes and females (in parentheses) are given on the top of each column. *Significant difference between WT and Rb5 or RBF ovaries at *P* < 0.05 by χ^2^-test.

We compared MPI progression in Rb5 and RBF ovaries to WT BALB/c ovaries by recording the percentage of oocytes at progressive MPI substages from 15.5 to 17.5 days postcoitum (dpc). Immunofluorescence (IF)-staining of SYCP3 in microspread chromosomes revealed localization of the synaptonemal complex axial elements (Fig. 1a). Many short thread-like SYCP3 stretches appeared at the early leptotene stage and SYCP3 stretches gradually elongated with further progression to the late leptotene stage. The zygotene stage was defined by the initiation and progression of pairing and synapsis between two chromosomes, which were seen as increasing number and length of thick SYCP3 stretches. The pachytene stage was characterized by complete synapses between homologous chromosomes, showing 20 thick linear SYCP3 stretches. To distinguish C- and D-telomeres, we used IF-staining of telomeres with anti-TRF1-antibody and centromeres with CREST human autoantibody. TRF1 foci with adjacent CREST foci identified C-telomeres, while those without CREST foci marked D-telomeres. RbT centromeres were identified by CREST foci without adjacent TRF1 foci. Because more chromosomes synapsed as MPI progressed, the number of D-telomere and C-telomere foci in a nucleus decreased from the initial 40 and 36 to a final 20 and 18, respectively, in Rb5 oocytes and from 40 and 28 to 20 and 14 in RBF oocytes. As to RbT centromeres, the number decreased from 2 to 1 in Rb5 oocytes and from 6 to 3 in RBF oocytes. In some of Rb5 and RBF oocytes, RbT centromeres were synapsed at late zygotene stage like the rest of chromosomes, suggesting that the definition of MPI substages in WT oocytes can be applied to RbT oocytes.

As summarized in Fig. 1b, the percentages of oocytes at progressive MPI substages were comparable among the ovaries of three genotypes at 15.5 dpc, suggesting that oocytes had entered meiosis around the same developmental stage. At 16.5 dpc, while 38.5% and 40.6% of oocytes in WT ovaries had reached the late zygotene and pachytene stages, respectively, smaller percentages of oocytes were seen at the late zygotene (24.1% and 29.2%) or pachytene (25.4% and 13.6%) stage in Rb5 and RBF ovaries. At 17.5 dpc, 77% of oocytes reached the pachytene stage in WT ovaries, whereas only 61.8% and 59.2% of oocytes did so in Rb5 and RBF ovaries, respectively. These results indicate a delay in MPI progression in both Rb5 and RBF ovaries compared to WT ovaries (*P* < 0.05 by χ^2^-test). The delay was more prominent in RBF ovaries than Rb5 ovaries, but no statistical difference was found between them.

### Distinct patterns in C- and D-telomere clustering and resolution

**Figure 2:**
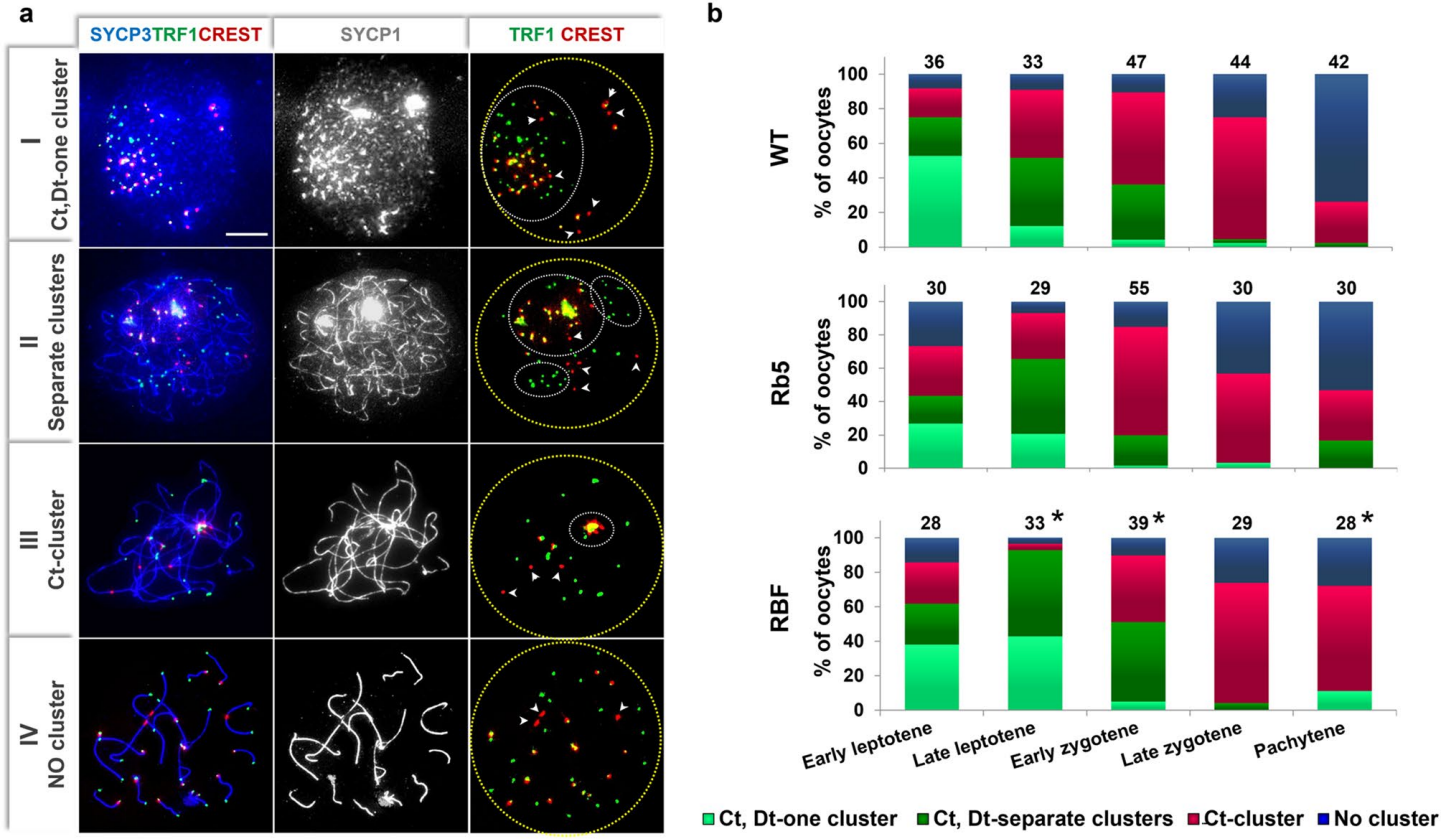
C- and D-telomere clustering and resolution in Rb5, RBF, and WT BALB/c mouse oocytes. (**a**) Representative images showing four types of telomere clustering in RBF oocytes. Telomere clusters are indicated by white broken line circles. RbT-centromeres are marked with arrowheads. Scale bar 10 μm. (**b**) Percentages of oocytes having each type of telomere clusters at progressive MPI substages. The total number of examined oocytes is given on the top of each column. Four females were examined in each strain. *Significant difference between RBF and WT or Rb5 oocytes at *P* < 0.05 by χ^2^-test.

We previously reported that C- and D-telomeres assume different patterns of clustering and resolution during MPI progression in WT BALB/c oocytes^40^. Here, we examined more WT oocytes and compared them with the C- and D-telomere clustering in Rb5 and RBF oocytes (examples in Fig. 2a) to determine whether the presence of metacentric RbT chromosomes affects the formation and resolution of telomere clusters. To distinguish clustering from random distribution, we used the “spatial statistics plugin” in the Image J software as previously described^40^. The pattern of telomere clustering in each nucleus was categorized as: (I) one cluster of C- and D-telomeres together; (II) separate clusters of C- and D-telomeres; (III) C-telomere cluster only and dispersed D-telomeres; and (IV) no cluster. Figure 2b summarizes the percentages of oocytes displaying each type of telomere clustering at progressive MPI substages. In WT ovaries, the clustering pattern gradually shifted from Type I to II, III and IV. For example, Type I cluster (C- and D-telomeres together) was found in 53% of oocytes at the early leptotene stage, whereas Type II (separate clusters of C-and D-telomeres) and Type III (only cluster of C-telomeres) were found each in 40% of oocytes at the late leptotene stage. Type III dominated the oocytes at early and late zygotene stages, whereas Type IV (no cluster) was found in 75% of oocytes at the pachytene stage.

In Rb5 ovaries, only 27% of early leptotene oocytes showed a Type I cluster, smaller than the percentage in WT ovaries. Otherwise, a similar shift from Type I to II, III and IV was observed (no significant difference). In RBF ovaries, the percentages of oocytes with four types of telomere clustering were comparable with the ovaries of other genotypes at the early leptotene stage. However, Type I and II dominated the oocytes at the late leptotene stage and Type II was still seen in 47% of oocytes at the early zygotene stage. Thus, the shift to Type III (resolution of D-telomere clustering) was delayed in RBF ovaries compared to WT or Rb5 ovaries (*P* < 0.05 by χ^2^-test). Ultimately, Type III (only cluster of C-telomeres) dominated RBF oocytes at the late zygotene stage and persisted in 74% of RBF oocytes at the pachytene stage, unlike WT or Rb5 oocytes (*P* < 0.05 by χ^2^-test). These results indicate that RBF oocytes retained the clusters of C- and D-telomeres until later MPI substages than WT oocytes.

While we analysed the telomere clustering patterns, we also monitored the position of RbT-centromeres (Fig. 2a). Of the 157 RBF oocytes analyzed, 34 (= 21.7%) showed four or more RbT-centromeres overlapped with the C-telomere cluster; six RbT-centromeres in 18 oocytes, five in 8 oocytes, and four in 8 oocytes. The remaining 123 oocytes (= 78.3%) showed all RbT-centromeres away from the C-telomere cluster; 47 (= 29.9%) showed RbT-centromeres clustered among themselves and 76 (= 48.4%) showed dispersed RbT-centromeres. When C-telomeres remained in clusters at the pachytene stage (n = 18), RbT-centromeres were always dispersed and away from the C-telomere cluster. Thus, RbT-centromeres behaved distinctly from C-telomeres throughout MPI progression.

### Preferential initiation of chromosome synapsis at D-telomeres

**Figure 3:**
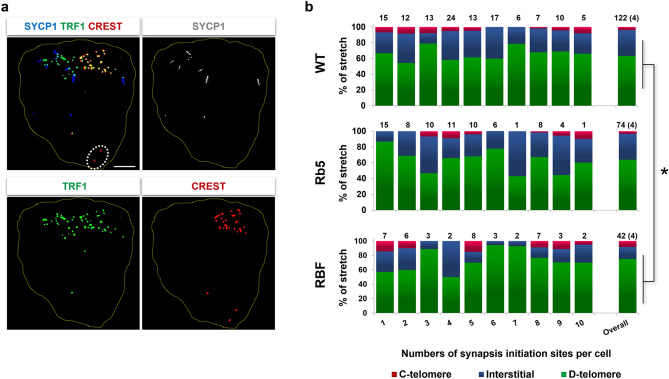
Initiation of chromosome synapsis in Rb5, RBF, and WT BALB/c oocytes at the early zygotene stage. (**a**) Rb5 oocyte with six short SYCP1 stretches (1–4 µm). C-telomeres, D-telomeres and interstitial regions are distinguished with IF-staining with anti-TRF1 and CREST antibodies. The merged image is followed by SYCP1, TRF1, and CREST staining alone. The circle with broken line indicates RbT-centromeres. (**b**) Percentages of short SYCP1 stretches at three chromosomal regions from the oocytes of three genotypes. The number of short stretches per oocyte is given on the X axis. The total number of examined oocytes is given on the top of each column. The results are combined as total at the right end. Four females were examined in each strain. *Significance in RBF oocytes compared to WT oocytes at *P* < 0.05 by χ^2^-test.

We compared the initiation of chromosome synapsis in Rb5 and RBF oocytes with that in WT oocytes by IF-staining for the synapsis-specific SYCP1, as well as TRF1 and CREST (Fig. 3a). We assumed that a short SYCP1 stretch (1–4 μm) captures the initial phase of synapsis, particularly when longer stretches are not yet seen. Here, we selected the oocytes at the early zygotene stage containing up to 10 SCYP1 short stretches, and determined whether each SCYP1 stretch was located at C-telomeric, interstitial or D-telomeric regions. Figure 3b summarizes the results. In the oocytes of all three genotypes, synapsis initiation was observed most frequently at D-telomeres, followed by interstitial regions, and rarely at C-telomeres. Since no major difference was seen in the results separately for the number of SYCP1 short stretches per oocyte, we pooled the data (shown at the right end). There was a stronger preference in the synapsis initiation at D-telomeres in RBF oocytes compared to WT oocytes (*P* < 0.05 by χ^2^-test). It should be noted that synapsis initiation was never seen at RbT-centromeres in the oocytes examined in this series.

### Frequency of meiotic chromosome crossovers

**Figure 4:**
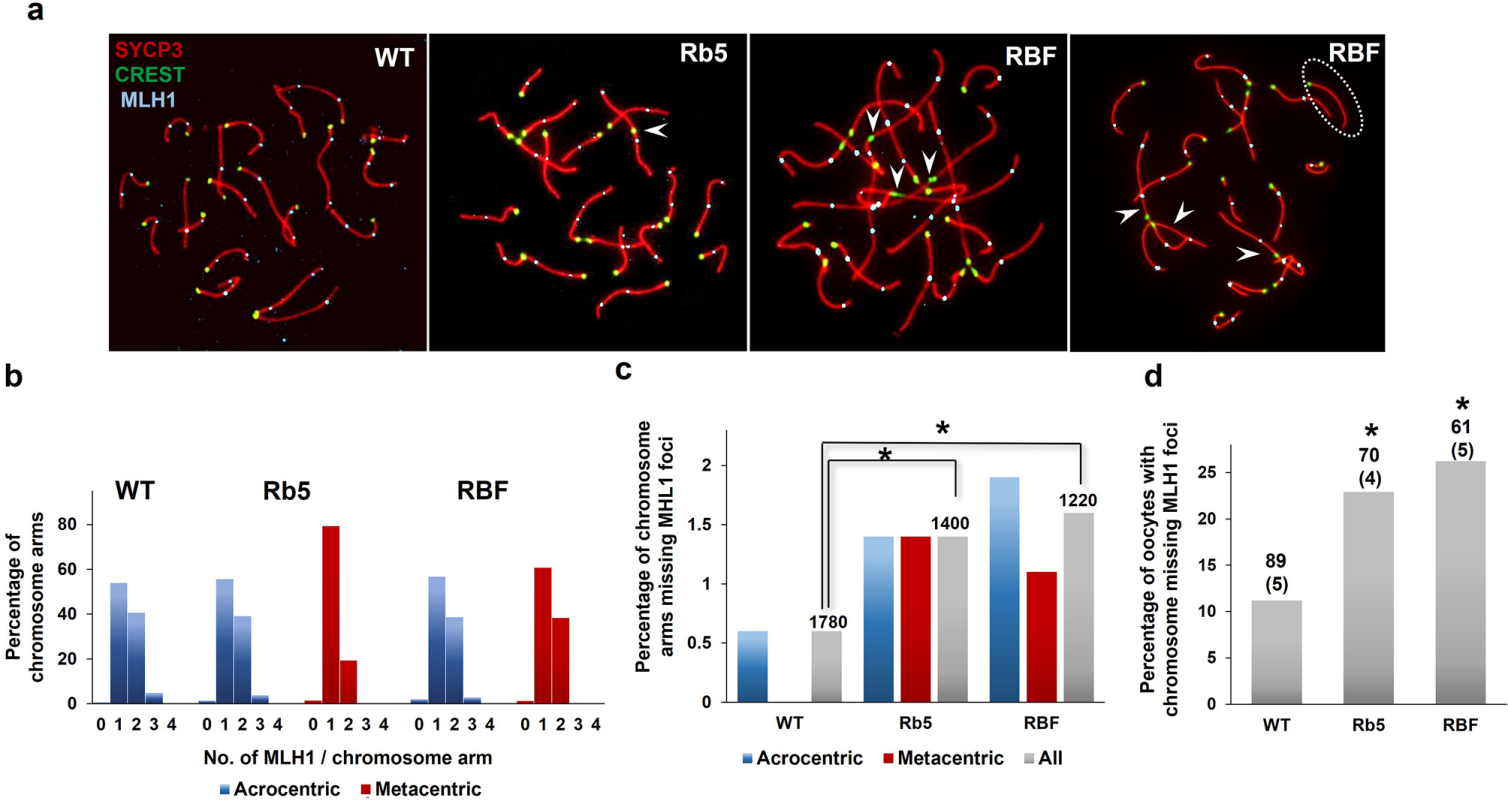
Crossover sites in Rb5, RBF, and WT BALB/c oocytes. (**a**) Crossover sites marked with MLH1 IF-staining in oocytes of each genotype. Arrowheads indicate RbT chromosomes. White circle with broken line indicates a chromosome lacking MLH1 foci in an RBF oocyte. (**b**) Histogram of the number of either acrocentric (blue) or metacentric (red) chromosome arms with 0–4 MLH1 foci in the oocytes of each genotype. (**c**) Percentage of either acrocentric or metacentric chromosome arms lacking MLH1 foci in each oocyte. The combined results are shown in the “All” column. The total number of examined chromosome arms is given on the top of each column. (**d**) Percentages of oocytes with at least one chromosome pair lacking MLH1 foci. The total number of examined oocytes and females (in parentheses) are given on the top of each column. *Significant difference at *P* < 0.05 by χ^2^-test.

We evaluated homologous chromosome recombination by analyzing the number and localization of MLH1, which is localized to stabilize crossover sites, in Rb5, RBF, and BALB/c WT oocytes at the pachytene stage at 17.5 dpc. Using IF-staining with CREST, SYCP3 and MLH1 antibodies (Fig. 4a), we counted the number of MLH1 foci on each acrocentric or metacentric RbT chromosome arm as summarized in Supplemental Table S2. The total number of MLH1 foci per oocyte was significantly smaller in both Rb5 and RBF oocytes compared to WT oocytes (*P* < 0.05 by either t-test or Mann–Whitney test). We assumed that since RbT fusion does not alter much of the chromosome length, the efficiency of meiotic recombination would be reflected into the number of MLH1 foci per chromosome arm. Figure 4b shows a histogram of the number of chromosome arms with 0, 1, 2, 3, or 4 MLH1 foci in the oocytes of each genotype. Most chromosome arms of either acrocentric or metacentric chromosomes showed one or two MLH1 foci, except that one MLH1 focus dominated on the RbT chromosome arm in Rb5 oocytes. We then calculated the frequency of chromosome arms lacking MHL1 foci as summarized in Fig. 4c. We first analysed acrocentric and RbT chromosome arms separately in Rb5 and RBF oocytes, but as no difference was found, we combined the results. None of the RbT chromosomes was found to have both arms lacking MLH1 foci in this set of data. The frequency of chromosome arms lacking MLH1 foci was significantly higher in Rb5 (20 out of 1400) and RBF (20 out of 1220) oocytes than in WT oocytes (10 out of 1780) at *P* < 0.05 by χ^2^-test. Thus the overall frequency of MLH1 foci was decreased in the presence of RbT chromosomes. Since a minimum of one crossover per chromosome pair, not per chromosome arm, is required for proper meiotic segregation, we also estimated the percentage of oocytes carrying at least one chromosome pair lacking MLH1 foci (Fig. 4d). The results, 22.8% (= 16/70) and 26.3% (= 16/61), respectively, in Rb5 and RBF oocytes were significantly higher than 11.2% (= 10/89) in WT oocytes at *P* < 0.05 by χ^2^-test.

### Rate of aneuploidy in ovulated MII-oocytes

**Figure 5:**
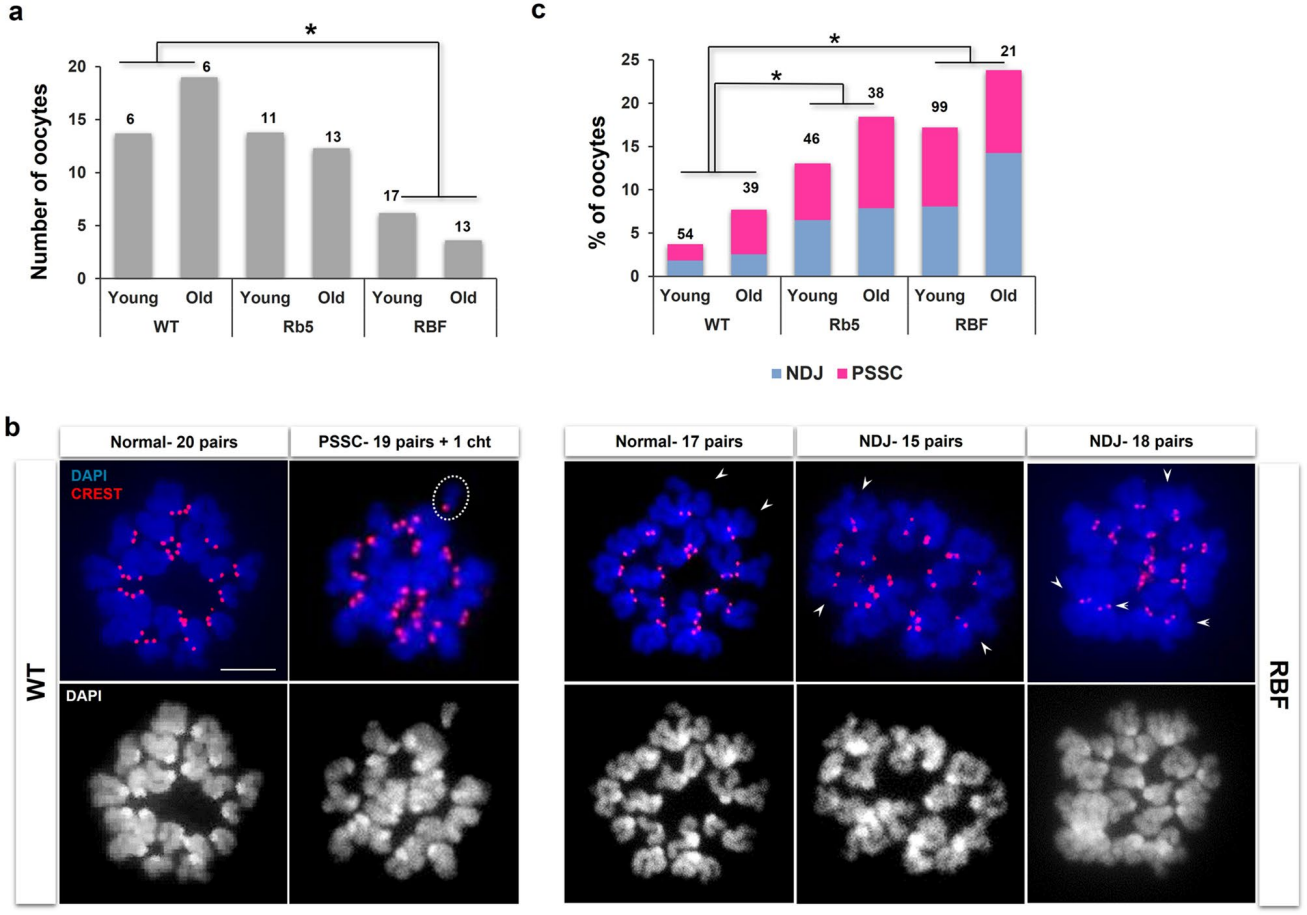
Aneuploidy in MII-oocytes ovulated by RbT-carrier and WT females. (**a**) The average number of ovulated oocytes by WT, Rb5, and RBF young (3–4 months) or old (6–9 months) females. The total number of examined mice is given on the top of each column. *Significant difference at *P* < 0.05 by Mann–Whitney test. (**b**) Representative images of nondisjunction (NDJ) and precocious separation of sister chromatids (PSSC) aneuploidy. White circle with broken line indicates a single chromatid (Cht) as the PSSC type aneuploidy. Arrowhead indicates an RbT chromosome. Scale bar 10 µm. (**c**) The rate of NDJ and PSSC aneuploidy in the oocytes from young vs. old females as shown in (**a**). The total number of examined oocytes is given on the top of each column. *Significant difference at *P* < 0.05 by χ^2^-test.

Oocytes containing chromosomes that lack meiotic crossover are prone to aneuploidy at the first meiotic division. To assess the aneuploidy rate in RbT-carrier and WT oocytes, MII-oocytes were collected after ovulation following gonadotrophin injections. To take the possible age effects on the aneuploidy rate into consideration, we examined MII-oocytes from young (3–4 months) and old (6–9 months) females separately. We first compared the number of ovulated oocytes between the two age groups of Rb5, RBF and WT females (Fig. 5a). Since we did not observe any significant difference between the two age groups, we combined the data, which showed a significantly smaller number from RBF females than WT females (*P* < 0.05 by Mann–Whitney test). We then examined aneuploidy in the MII-metaphase chromosomes spread from each oocyte, using CREST IF-staining and DAPI nuclear staining (Fig. 5b). Aneuploidy was categorized as nondisjunction (NDJ), meaning the gain or loss of a whole chromosome pair, or precocious separation of sister chromatids (PSSC), meaning the gain or loss of a sister chromatid instead of a chromosome (Supplemental Table S3). Since we did not observe any significant difference between the females of the two age groups (Fig. 5c), the results were combined for statistical analysis. The overall aneuploidy rate was significantly higher in RBF (18.3%) and Rb5 (15.5%) oocytes than in WT (5.4%) oocytes (*P* < 0.05 by χ^2^-test). NDJ- and PSSC-type aneuploidies were equally found in the oocytes of all genotypes. It must be noted that one Rb5 oocyte showed PSSC of the RbT chromosome whereas one RBF oocyte showed NDJ with an extra RbT chromosome (Fig. 5b). Thus, both acrocentric and RbT chromosomes appeared to be subject to aneuploidy.

## Discussion

Our study revealed that the presence of metacentric RbT chromosomes among acrocentric chromosomes negatively affected the progression of MPI and the frequency of homologous recombination and crossover in the mouse oocyte. More specifically, a larger number of chromosome arms lacked MLH1 foci, and a larger number of oocytes carried at least one chromosome lacking MLH1 foci in both Rb5 and RBF ovaries compared with WT ovaries. Subsequently, the aneuploidy rate was higher in the MII-oocytes ovulated by Rb5 or RBF females compared with those ovulated by WT females. These results agree with our prediction that the low aneuploidy rate of the mouse oocyte is due to the fact that all chromosomes are acrocentric.

In our results, MPI progression was delayed in RbT oocytes compared to WT oocytes. The onset of meiosis and early MPI progression appeared to be comparable among the ovaries of three genotypes. However, further MPI progression was delayed in RbT ovaries, concomitant with altered behaviors of telomeres; D- and C-telomeres remained in clusters in RBF oocytes until later MPI substages than those in WT oocytes. This trend was also seen in Rb5 oocytes although the difference did not reach statistical significance. Furthermore, the initiation of chromosome synapsis was more strongly biased towards D-telomeres in RBF oocytes compared to WT oocytes. We have previously suggested that D-telomeres may have a better chance to find their partners to synapse by resolving their clusters early while C-telomeres remain in clusters^40^. Therefore, we anticipated that in RBF oocytes, the persistent D-telomere clusters would interfere with their preferential synapsis. However, our current results did not agree with this prediction. Rather, the delayed resolution of D-telomeres may have given a disadvantage to the interstitial region to initiate synapsis. Our observation that C-telomere clusters persisted in most RBF oocytes at the pachytene stage suggests that the C-telomere resolution is not essential for the completion of chromosome synapsis. We cannot exclude the possibility that the genetic background of RBF mice contributed to these differences since no WT control on the same genetic background is available. However, similar delays in MPI progression and altered telomere behaviors were observed in Rb5 oocytes, albeit to milder extents, compared to WT oocytes that share the genetic background. We speculate that the physical presence of RbT chromosomes may interfere with the coordinated movement of acrocentric chromosomes.

Crossover between homologous chromosomes is the most important end point of MPI. Each crossover site can be visualized by IF-staining of MLH1, which corresponds to 90% of meiotic recombination^46^. The frequency of meiotic recombination generally depends on the chromosome length. Since the Robertsonian fusion does not much alter the length of chromosome arms, we first compared the number of MLH1 foci per chromosome arm in the oocytes of three genotypes. One or two MLH1 foci were detected consistently on either acrocentric or metacentric chromosome arms, except that one MLH1 foci dominated on the Rb5 metacentric chromosome arm. Nonetheless, the total number of MLH1 foci per oocyte was decreased and the percentage of chromosome arms lacking MLH1 foci was increased in Rb5 and RBF oocytes compared to WT oocytes. Both metacentric and acrocentric chromosome arms showed equal chance to lack MLH1 foci. These results suggest that the overall efficiency of meiotic recombination, which matured into crossovers, decreased in the presence of RbT chromosomes. Since one crossover site per chromosome pair should be sufficient to secure homologous chromosome segregation at the first meiotic division, we also compared the number of MLH1 foci per chromosome. Our results showed that a larger percentage of oocytes carried at least one chromosome pair lacking MLH1 foci in either Rb5 or RBF females compared to WT females. These results are consistent with the higher rates of aneuploidy in RbT oocytes.

Our current results in oocytes differ from the previous reports in spermatocytes carrying similar RbT chromosomes. Dumont et al.^47^ reported that spermatocytes of Rb5 and RBF male mice show higher frequencies of chromosome arms lacking MLH1 foci compared to BALB/c WT spermatocytes, in agreement with our results. However, they found that metacentric RbT chromosomes were more likely to lack MLH1 foci than acrocentric chromosomes, which was not observed in our study. By contrast, Capilla et al.^48^ did not find a significant difference in the number of chromosome arms lacking MLH1 foci among the RbT and WT mice caught in Spain. This discrepancy can be attributed to fundamental differences in meiotic recombination between spermatocytes and oocytes^49^.

The molecular mechanisms responsible for the interference of meiotic recombination by the presence of RbT chromosomes remain to be investigated. It has been suggested that the meiotic recombination sites are predetermined during the premeiotic S phase, prior to DSB formation, in mouse spermatocytes^50^. However, this hypothesis does not exclude a role for chromosome interaction in the processing of meiotic recombination during MPI progression. We speculate that the delay in homologous pairing and synapsis may have reduced the efficiency of recombination in RbT oocytes. The late resolution of C-telomeres at the pachytene stage in RBF oocytes, which was not observed in WT oocytes, may indicate that a longer time was required for completing homologous recombination in RbT oocytes. Conversely, the inefficient processing of meiotic recombination may have translated into a delay in MPI progression or C-telomere resolution.

During fetal and neonatal development, 70–80% of the initial oocyte population is eliminated to restrict the number of oocytes in the ovarian reserve^51–53^. The cause or mechanism of this major oocyte loss is not yet fully understood. Nonetheless, incomplete DSB repair and synaptic errors are the two known causes for oocyte surveillance^54–56^. Therefore, the meiotic recombination observed in the oocytes in fetal ovaries may not represent the oocytes in adult ovaries. In the present study, we examined the MII-oocytes ovulated by adult females after gonadotropin injections. The aneuploidy rates of 15% and 18% in the oocytes of Rb5 and RBF females, respectively, were significantly higher than the 5% in the oocytes of WT females. All these figures were lower than the percentages of oocytes carrying at least one chromosome pair lacking MLH1 foci (above). These differences can be explained by selective oocyte loss, efficiency of MLH1 immunostaining, or the fact that some homologous chromosomes without crossovers can segregate properly by random segregation. The MLH1-independent crossovers may also have contributed to the proper chromosome segregation^57,58^.

Since the aneuploidy rate is known to increase with maternal age, we initially analyzed the results in two age groups. Although we observed a consistent trend towards higher aneuploidy rate in the older age group of each genotype, the difference did not reach statistical significance. We did not examine the mice older than 9 months because RBF females tend to lose their fertility. We also distinguished NDJ- and PSSC-type aneuploidy, which were found equally in the oocytes of three genotypes and in two age groups. We conclude that the inefficient meiotic recombination and crossover during MPI progression resulted in higher aneuploidy rates in both Rb5 and RBF oocytes similarly up to 9 months of age.

The frequency and distribution of MLH1 foci and the aneuploidy rate have been extensively studied in human oocytes. Cheng et al.^59^ reported that 42 out of 176 pachytene oocytes (24%) showed at least one bivalent lcking MLH1 foci. However, the same team later examined 7396 oocytes from 160 fetal ovarian samples and found that only 7.3% carried chromosome pairs lacking MLH1 foci^18^. The authors explained this discrepancy partly by selection of oocytes for analyses. However, the aneuploidy rate among teen-agers is reported to be around 40%^6^. This discrepancy is difficult to reconcile. Although MLH1 foci mark crossover sites and can be conveniently visualized, immunostaining is prone to technical variability. In our study, we found a reasonable correlation between the frequency of MLH1 foci and the rate of aneuploidy in mouse oocytes. Furthermore, the oocytes of three genotypes were processed simultaneously, therefore the results should be independent of experimental irregularity. In humans, the rate of aneuploidy decreases to around 20% in young women and then increases again with maternal age over 35 years^6^. They found that chromosomes 1–5 (largest chromosomes) are more vulnerable to NDJ-type aneuploidy at young ages, which can be caused by a lack of crossovers in fetal life. By contrast, PSSC-type aneuploidy dominates the oocytes of older women, due to the deterioration of various chromosomal and cellular components such as sister chromatid cohesins and mitochondria with advanced age^6,16^. In our study, NDJ- and PSSC-type aneuploidy equally contributed to the higher aneuploidy rates in Rb5 and RBF oocytes than WT oocytes. Although we arbitrarily separated two age groups in mice, both may correspond to the peak reproductive age range in women.

In theory, a higher rate of aneuploidy in MII-oocytes may result in a smaller litter size. However, no statistical difference was found between BALB/c and Rb5 females in our records. The number of fetuses per litter was 7.9 ± 3.1 (Mean ± SD, n = 11) vs. 7.8 ± 3.2 (n = 13) at 15.5—17.5 dpc. The number of pups at delivery (19.5—20.5 dpc) was 7.1 ± 3.5 (n = 13) vs. 5.8 ± 2.4 (n = 9), respectively. Since we induced ovulation by gonadotropin injections in our study, the total number of oocytes obtained from each female was much larger. How aneuploidy oocytes contribute to fertilization or embryonic development in these mouse strains remain to be examined.

## Materials and methods

### Mouse

BALB/cByJ (BALB/c), CByJ.RBF-Rb(8.12)5Bnr (Rb5), and RBF/DnJ(1.3; 8.12; 9.14) (RBF) mice were purchased from the Jackson Laboratory (Bar Harbor, ME) and maintained in homozygosity in our mouse colony. Female mice were left with males of the same strain overnight and the noon of the day when the vaginal plug was found was defined as 0.5 dpc. All animal procedures were performed in accordance with the Canadian Council on Animal Care and approved by the McGill University Animal Care Committee. All studies involving animals are reported in accordance with the ARRIVE guidelines (https://arriveguidelines.org).

### Microspread ovarian cell preparations

Microspread ovarian cells on glass slides were prepared as previously described^60^. In brief, ovaries isolated from fetuses at 15.5–17.5 dpc were individually dissociated into single cells after sequential digestion with 0.05% collagenase and 0.125% trypsin in siliconized microfuge tubes. The dissociated cells were spun down, and dispersed into a hypotonic solution (0.45% NaCl, pH 8.0) in chambers over histology slides for 10–12 min, followed by centrifugation and fixation in 1% paraformaldehyde (pH 8.0) twice and washing in 4% PhotoFlow (Kodak) thrice. The slides were dried under vacuum and kept with Silica gel in sealed boxes at − 20 °C until use.

### Immunofluorescence (IF) staining

Microspread ovarian cells on histology slides were washed three times each for 10 min in Holding Buffer containing normal goat serum, bovine serum albumin and Triton X-100^60^. The slides were then incubated with primary antibodies overnight at room temperature, incubated with secondary antibodies conjugated with either biotin or fluorescent dyes for 1 h at 37 °C, and incubated with avidin conjugated with fluorescent dyes of assorted colors for 30 min at 37 °C. All primary and secondary antibodies used are listed in Supplemental Table S1. After IF-staining, the slides were washed, air-dried, and mounted in the Prolong Gold Antifade mounting medium containing 4′,6-diamidin-2′-phenylindole dihydrochloride (DAPI) (Molecular Probe, Eugene, OR). Fluorescent signals were captured and analyzed under an epifluorescence microscope (Leica Microscope System DM6000B, Germany).

### Telomere clustering analysis

To analyze the D- and C-telomere clustering, we used the “spatial statistics plugin” program in the ImageJ software, as we have previously described^40,61^. The “plugin” determines a point pattern (positions of objects of interest) distributed within a reference structure. This pattern is determined based on two cumulative distribution functions (CDF); F-function and G-function. In the G-function, the cumulative distance between the objects and their nearest neighbors is computed, while in the F-function, the distance between a typical position within the reference structure and its nearest objects in the pattern is calculated. The “plugin” also creates several examples with random point distribution and compare the CDF with that of the sample of interest to evaluate whether the distribution in the sample is statistically deviate from the random distribution. At the end, it gives the normalized F and G functions with the values between 0 and 1; F = 1–0.95 and G = 0–0.05 is expected for clustered pattern, F = 0–0.05 and G = 1–0.95 for regular pattern (which is not the case in our experiments), and F and G between 0.05 and 0.95 or random pattern^61^.

### Aneuploidy analyses in MII oocytes

Female mice at 3–4 months (young) or 6–9 months (old) of age were intraperitonially injected with 5 IU equine chorionic gonadotrophine (eCG, SIGMA) and human CG (hCG, SIGMA) with a 48 h interval. Oocytes were collected from the oviducts at 14 h post-hCG injection. After zona pellucida was dissolved with acid Tyrode^62^, chromosomes were spread onto histology slides and labeled with CREST antibody, followed by the second antibody, and mounted in the Prolong Gold Antifade mounting medium containing DAPI. Chromatid numbers and the type of aneuploidy as non-disjunction or precautious separation of sister chromatid, if present, were determined under the epifluorescence microscope.

### Statistic analyses

In all experiments, microspread cells were prepared from an ovary each from at least three females and two litters. In all experiments, data were evaluated by χ^2^-test, except that the comparison of the numbers of ovulated MII oocytes were evaluated by the Mann–Whitney test using Graphpad Prism 6.01.

## Supplementary Information

Supplementary Tables.
