# Supplementary material for: Mouse oocytes carrying metacentric Robertsonian chromosomes have fewer crossover sites and higher aneuploidy rates than oocytes carrying acrocentric chromosomes alone
Source: Sci Rep. 2022 Jul 14;12:12028. doi: 10.1038/s41598-022-16175-6 (PMC9283534; doi:10.1038/s41598-022-16175-6)
Supplement: Supplementary file 1 — Supplementary Tables. [file 41598_2022_16175_MOESM1_ESM.pdf]

Supplemental materials

Mouse oocytes carrying metacentric Robertsonian chromosomes have fewer crossover sites and higher aneuploidy rates than oocytes carrying acrocentric chromosomes alone

Parinaz Kazemi,[1] Teruko Taketo[1,2,3 *]

[1] Department of Biology, McGill University, Montreal, QC, H3A 1B1, Canada

[2] Department of Surgery, McGill University, RI-MUHC, Montreal, QC, H4A 3J1, Canada

[3] Department of Obstetrics/Gynecology, McGill University, RI-MUHC, Montreal, QC, H4A 3J1, Canada

*Corresponding author: e-mail, teruko.taketo@mcgill.ca

Table S1. Primary and secondary antibodies used for immunofluorescence staining

	Antibody	Host Species	Company	Catalog Number	Concentration
Primary	CREST	Human	ImmunoVision	HCT-0100	1:1000
	SYCP3	Rabbit	Abcam	Ab15093	1:500
	SYCP1	Rabbit	Abcam	Ab97672	1:500
	TRF1	Mouse	Agrisera	AS163961	1:500
	MLH1	Mouse	BD Pharmigen	550838	1: 500
Secondary	Anti-human- FITC	Goat	PIERCE	31528	1:1000
	Anti-mouse- Alexa Fluor 647	Goat	Invitrogen	A-21236	1:1000
	Anti-rabbit biotin	Goat	PIERCE	31823	1:1000
	Streptavidin-RRX		Invitrogen	S6366	1:1000

Table S2. The number of MLH1 foci in the pachytene oocytes from WT BALB/c, Rb5, or RBF females.

	No. of oocytes	Total No. of MLH1 foci/oocyte (Mean ± SD)	No. of oocytes with chromosome lacking MLH1 foci	Chromosome type	No. of chromosome arms	No. of MLH1 foci /chromosome arm				
						**0**	**1**	**2**	**3**	**4**
**WT**	89	30.5 ± 1.6	10	**Acrocentric**	1780	10	959	722	84	5
**Rb5**	70	28.4 ± 0.76*	16	**Acrocentric**	1260	18	701	492	47	2
				**Metacentric**	140	2	111	27	0	0
				**All**	1400	20	812	519	47	2
**RBF**	61	28.7± 0.6*	16	**Acrocentric**	854	16	484	329	24	1
				**Metacentric**	366	4	222	140	0	0
				**All**	1220	20	706	469	24	1

* difference from WT at $P<0.05$ by either t-test or Mann-Whitney test.

Table S3. The rate of NDJ and PSSC aneuploidy in the MII-oocytes ovulated by WT BALB/c, Rb5, or RBF females.

**Genotype**	**No. of oocytes**	**NDJ**		**PSSC**	
		**Gain**	**Loss**	**Gain**	**Loss**
**WT**	93	0	2	0	3
**Rb5**	84	1	5	2	5
**RBF**	120	3	8	5	6
